# The telomere lengthening conundrum – it could be biology

**DOI:** 10.1111/acel.12555

**Published:** 2016-12-12

**Authors:** Melissa Bateson, Daniel Nettle

**Affiliations:** ^1^ Institute of NeuroscienceNewcastle UniversityHenry Wellcome Building, Framlington PlaceNewcastle upon TyneNE2 4HHUK

**Keywords:** computational model, leucocyte telomere length, measurement error, telomere attrition, telomere dynamics, telomere lengthening

## Abstract

Longitudinal studies of human leucocyte telomere length often report a percentage of individuals whose telomeres appear to lengthen. However, based on theoretical considerations and empirical data, Steenstrup *et al*. (Nucleic Acids Research, 2013, vol 41(13): e131) concluded that this lengthening is unlikely to be a real biological phenomenon and is more likely to be an artefact of measurement error. We dispute the logic underlying this claim. We argue that Steenstrup *et al*.'s analysis is incomplete because it failed to compare predictions derived from assuming a scenario with no true telomere lengthening with alternative scenarios in which true lengthening occurs. To address this deficit, we built a computational model of telomere dynamics that allowed us to compare the predicted percentage of observed telomere length gainers given differing assumptions about measurement error and the true underling dynamics. We modelled a set of scenarios, all assuming measurement error, but both with and without true telomere lengthening. We found a range of scenarios assuming some true telomere lengthening that yielded either similar or better quantitative fits to the empirical data on the percentage of individuals showing apparent telomere lengthening. We conclude that although measurement error contributes to the prevalence of apparent telomere lengthening, Steenstrup *et al*.'s conclusion was too strong, and current data do not allow us to reject the hypothesis that true telomere lengthening is a real biological phenomenon in epidemiological studies. Our analyses highlight the need for process‐level models in the analysis of telomere dynamics.

## Introduction

In adult humans, cross‐sectional and longitudinal studies of telomere dynamics show that, on average, leucocyte telomere length (TL) declines at a rate of 20–45 base pairs (bp) per year (Vaziri *et al*., 1993; Slagboom *et al*., 1994; Gardner *et al*., 2005; Aviv *et al*., 2009; Ehrlenbach *et al*., 2009; Farzaneh‐Far *et al*., 2010; Chen *et al*., 2011; Kark *et al*., 2012; Steenstrup *et al*., 2013b; Bendix *et al*., 2014). While age‐dependent telomere attrition is the average result, in longitudinal studies leucocyte TL is often observed to lengthen over time in a percentage of individuals ranging from 0 to 50% of the sample (Gardner *et al*., 2005; Aviv *et al*., 2009; Ehrlenbach *et al*., 2009; Nordfjäll *et al*., 2009; Farzaneh‐Far *et al*., 2010; Chen *et al*., 2011; Svenson *et al*., 2011; Kark *et al*., 2012; Shalev *et al*., 2013; Steenstrup *et al*., 2013b). This telomere lengthening is potentially exciting, because it suggests that a key marker of cellular aging is sometimes reversible and that this can be detected in longitudinal studies of humans. However, current methods for measuring telomere length are far from precise. Technical variation, measured as the intra‐ or interassay coefficient of variation (CV), has been estimated at 1.4–9.5% within laboratories (Martin‐Ruiz *et al*., 2015). This CV translates into a standard deviation of 98–665 bp for a mean TL of 7000 bp, which is considerable in comparison with the annual attrition rates cited above. An important implication of measurement error is that repeated measures of the same sample are unlikely to be identical, and assuming that measurement error is unbiased and symmetrically distributed, on average 50% of second TL measurements on the same sample will be larger than the first. It follows that if measurement error is present, there is a nonzero probability that a proportion of individuals in a longitudinal study will be incorrectly classified at follow‐up as TL gainers. The conclusion is that observed telomere lengthening in longitudinal studies could simply be an artefact of measurement error (Steenstrup *et al*., 2013a,b; Verhulst *et al*., 2015).

**Figure 1:**
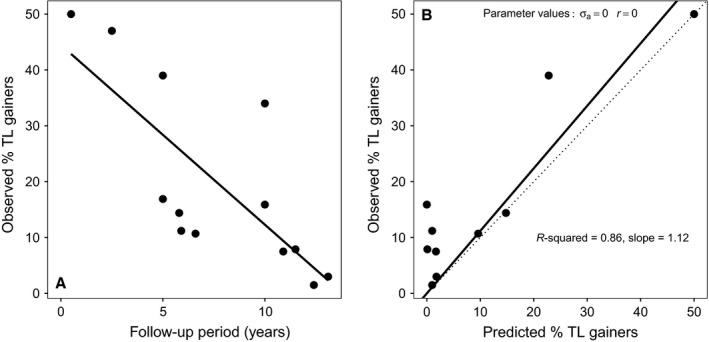
The evidence presented by Steenstrup *et al*. (2013a) to support their claim that observed telomere lengthening is predominantly, if not entirely, an artefact of measurement error exacerbated by short follow‐up periods. (A) The predicted negative relationship between follow‐up period and percentage of TL gainers (*n* = 13 studies). The solid line shows the best‐fitting linear regression. (B) Agreement between theoretical predictions based on assuming constant telomere attrition and measurement error, and empirically observed percentage of TL gainers (*n* = 10 studies). The dotted line has a slope of 1 and shows the expectation if predictions were perfect. The solid line shows the best‐fitting line obtained from regression through the origin; the r‐squared value and slope of this line are given on the graph.

In an influential recent paper, Steenstrup *et al*. (2013a) attempted to explore the empirical support for a measurement error‐based account of observed telomere lengthening. They did this by assuming a specific scenario for the processes underlying telomere dynamics and measurement error in order to generate theoretical predictions that could be compared with empirical data obtained from published studies. For telomere dynamics, they assumed that all individuals exhibit age‐dependent telomere attrition with a constant rate of telomere loss per year (i.e. no variation in the rate of telomere attrition and no true TL gainers). They further assumed that TL at baseline and follow‐up is measured with error and that measured TLs are normally distributed around a mean equal to the true TL with a standard deviation determined by the precision of the assay (expressed as interassay CV). Following from these assumptions, Steenstrup *et al*. (2013a) made two predictions that could be tested against published empirical data from longitudinal studies of TL. First, they predicted that there should be a negative relationship between the follow‐up period and the percentage of TL gainers observed in a study. Second, they predicted the exact percentage of TL gainers due to measurement error expected in each empirical study. In both cases, their predictions fitted fairly well with the observed data on percentage of TL gainers from 13 published studies (Fig. 1). They did note that the observed percentage of TL gainers was somewhat underpredicted by their simple model (Fig 1B), but attributed this underprediction to the absence of any variation in the rate of telomere attrition in their model (as opposed to their assumption that true telomere lengthening does not occur). On the basis of their findings, Steenstrup *et al*. (2013a) concluded that the apparent telomere lengthening observed in longitudinal studies is not a real biological phenomenon but ‘*is predominantly, if not entirely, an artefact of measurement error,… exacerbated by short follow‐up periods’*.

Steenstrup *et al*. (2013a) paper has had a substantial impact on the telomere dynamics literature since its publication. It has been cited in methodological papers to urge caution in the interpretation of longitudinal TL data (e.g. Nussey *et al*., 2014; Verhulst *et al*., 2015; Bateson, 2016) and in empirical studies to cast doubt over whether observed telomere lengthening represents a real biological phenomenon (e.g. Shalev *et al*., 2014; Carulli *et al*., 2016).

However, Steenstrup *et al*. (2013a) argument is based on a logically invalid inference. Their argument is of the general form: ‘If P then Q; Q therefore P’. Translating this into words, they argue: ‘If telomeres do not really lengthen and all observed lengthening is due to measurement error, then we should observe a given percentage of individuals displaying apparent telomere lengthening in longitudinal studies; we empirically observe approximately this predicted percentage of individuals displaying lengthening; therefore telomeres do not really lengthen’. In formal logic, this is an error of reasoning known as affirming the consequent or the fallacy of the converse. It is invalid because demonstrating that P implies Q and demonstrating that Q is true do not allow the conclusion that P is true. This is because many different scenarios (including P, but also other possibilities) might lead to an observation of Q, meaning that observing Q provides no information about the truth of P. In their analysis, Steenstrup *et al*. (2013a) consider only a single scenario of age‐dependent constant‐rate telomere attrition (i.e. no true telomere lengthening) assessed with measurement error. Although the observed data are predicted fairly well using these assumptions (Fig. 1), unless the predictions of alternative accounts of telomere dynamics, specifically scenarios including some true telomere lengthening, are compared with the scenario of no true lengthening, then it is incorrect to conclude that the observed empirical data support the truth of a scenario with no true lengthening. Thus, in order to test properly the hypothesis that observed telomere lengthening is an artefact of measurement error, it is necessary to additionally compare the predictions made in scenarios that include true telomere lengthening. Only if scenarios with no true lengthening are found to predict the observed data better than scenarios with true lengthening can Steenstrup *et al*.'s (2013a) conclusions be upheld. Alternatively, if scenarios involving true telomere lengthening are found to predict the observed data either equally well or better than Steenstrup *et al*.'s (2013a) model, this would suggest that it is not possible to reject the hypothesis that true telomere lengthening is contributing to observed cases of measured telomere lengthening.

In the current paper, we used a computational approach to create a theoretical model of underlying true telomere dynamics with which it is possible to generate a range of different scenarios concerning whether any true telomere lengthening occurs and what the characteristics of this lengthening are. We did this by varying two parameters affecting true telomere dynamics. To model the possibility of telomere lengthening in a proportion of individuals, we included a parameter (σ_*a*_) that determines the standard deviation of the telomere attrition experienced in each year. This parameter allowed us to model the empirical observation that there is substantial variation in observed annual telomere attrition (e.g. Steenstrup *et al*., 2013b; Puterman *et al*., 2015). By sampling annual attrition from a normal distribution, we allowed for the possibility that annual attrition could sometimes be negative (i.e. that there was true telomere lengthening). To model the possibility that the rate of telomere change might be autocorrelated in successive years within individuals, we included a parameter (*r*) that determines the size of this autocorrelation. This second parameter allowed us to model the empirical observation that there are substantial individual differences in the pace of aging (Belsky *et al*., 2015). We used the resulting model to explore how the presence of true telomere lengthening and added measurement error affect the predicted percentage of apparent TL gainers observed in a longitudinal study. We specifically set out to answer two questions directly inspired by the results in Steenstrup *et al*.'s (2013a) paper. First, is the prediction of a negative relationship between follow‐up period and observed percentage of TL gainers identified by Steenstrup *et al*. unique to a scenario in which TL declines at a constant rate with time and is measured with error? Second, does Steenstrup *et al*.'s assumption of no true telomere lengthening provide the best quantitative fit to the observed data on the percentage of TL gainers?

## Results

### Simulations produce scenarios with differing telomere dynamics

**Figure 2:**
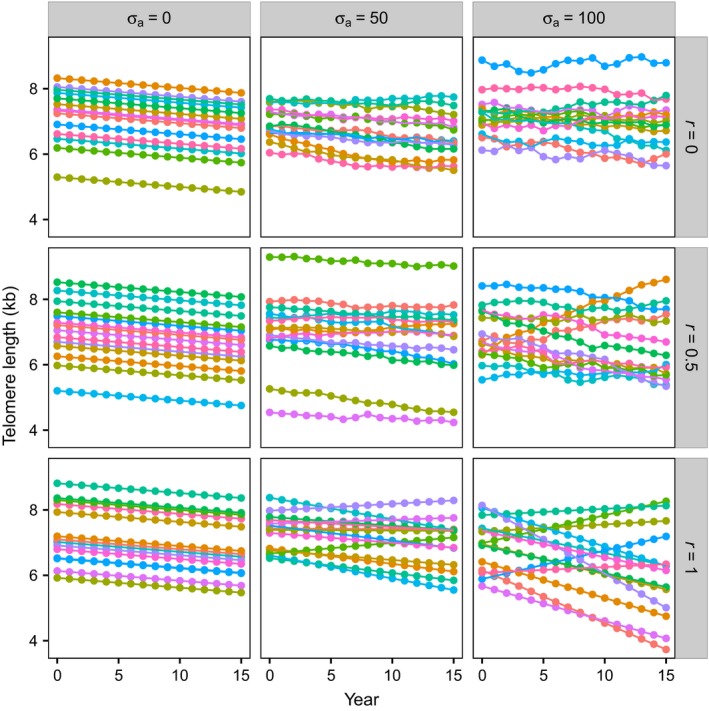
Simulations produce scenarios with differing telomere dynamics. Panels show examples of the true TL data (i.e. without added measurement error) produced by the computational model using the default values given in Table 2. True TL is plotted as a function of year for the nine scenarios obtained by combining three values of the standard deviation of annual attrition, σ_*a*_ (columns), with three values of the autocorrelation of annual attrition, *r* (rows). For clarity, each panel shows TL data from only 15 randomly chosen individuals from the 10 000 in each simulation.

Figure 2 illustrates how true TL changes over time for a random sample of 15 individuals taken from runs of the simulation with three values of σ_*a*_ (columns) and three values of *r* (rows). The left‐hand column demonstrates the scenario in which there is no variation in the annual rate of attrition (σ_*a*_ = 0) and all individuals’ telomeres shorten at the same, constant rate (the default attrition assumed was 30 bp year^−1^); the value of *r* has no effect if there is no variance in annual attrition, and thus, all three panels in this column simulate the same dynamics. The telomere dynamics when σ_*a*_ = 0 reproduce those assumed by Steenstrup *et al*. (2013a). The middle and right‐hand columns of Fig. 2 demonstrate the effects of increasing the standard deviation in the annual rate of attrition; because the rate of attrition can now be negative as well as positive, some individuals show increases in TL. The bottom row demonstrates the effect of setting the autocorrelation between rate of attrition in successive years to one; this generates a constant rate of attrition within individuals, but variation in rate of attrition between individuals, with a small proportion of individuals consistently gaining TL when σ_*a*_ > 0.

The mean annual rate of attrition μ_*a*_ was set to 30 bp year^−1^ for all simulations. As expected from the properties of Eqn (1), all of the scenarios modelled show a mean decrease of 30 bp year^−1^ (Fig. S1A, Supporting information). When there is no variation in the annual rate of attrition (σ_*a*_ = 0), the standard deviation of TL in a year remains constant over time, but when the variation in annual attrition is nonzero (σ_*a*_ > 0), this standard deviation increases with the duration of the follow‐up period and the size of the standard deviation in annual attrition (σ_*a*_; Fig. S1B, Supporting information).

### Observed percentage of TL gainers always declines with follow‐up period

**Figure 3:**
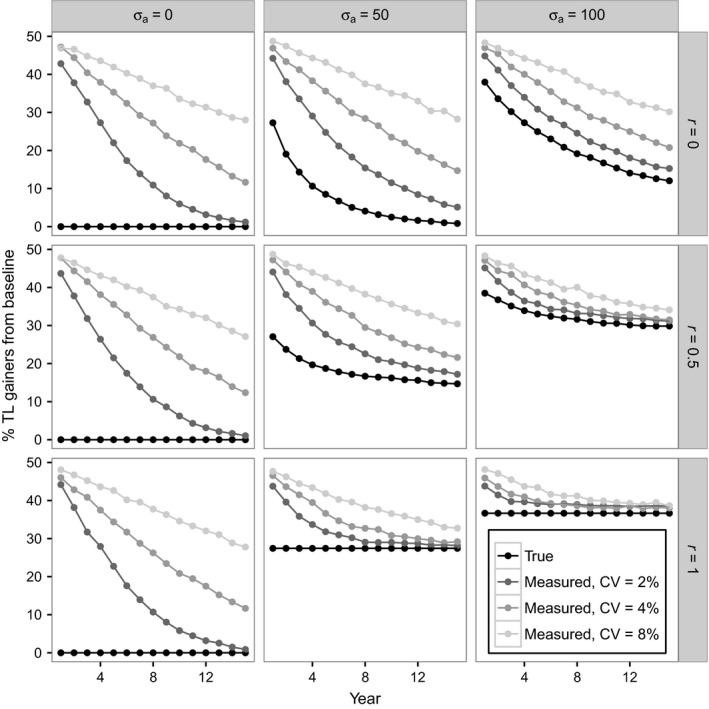
Observed percentage of TL gainers always declines with follow‐up period independent of the telomere dynamics assumed. Panels show percentage of individuals showing a gain in TL from baseline in each follow‐up year for the same nine scenarios depicted in Fig. 2. The black lines show the percentage of true TL gainers from baseline. The grey lines show the percentage of observed TL gainers from baseline given different levels of measurement error at baseline and follow‐up (*CV*s of 2, 4 and 8%). Simulations assumed the default values in Table 2; TL was ‘measured’ only once at each time point.

Figure 3 shows the percentage of TL gainers relative to baseline as a function of follow‐up time for the same nine scenarios of telomere dynamics depicted in Fig. 2. The black lines in Fig. 3 show the percentage of true TL gainers in each follow‐up year, whereas the grey lines show the percentage of TL gainers observed in each follow‐up year when measurement error is added (*CV* = 2, 4 and 8%). The left‐hand column of Fig. 3, in which there is no variance in annual telomere attrition, reproduces the result in Fig. 1A that was predicted and observed by Steenstrup *et al*. (2013a): there is a negative relationship between follow‐up period and observed percentage of TL gainers when measurement error is assumed, despite the absence of any true TL gainers. However, the middle and right‐hand columns of Fig. 3 show that when measurement error is added (*CV* > 0), there is always a negative relationship between follow‐up period and percentage of TL gainers, even when a nonzero percentage of individuals show true telomere lengthening. Furthermore, even if measurement error is zero, a negative relationship between follow‐up period and the percentage of true TL gainers is observed, as long as the autocorrelation in annual attrition is not perfect (i.e. *r *<* *1). Therefore, the negative relationship observed by Steenstrup *et al*. (2013a; Fig. 1A) is not a unique prediction of assuming a scenario of telomere dynamics in which there are no true gainers and telomeres are measured with error. Indeed, for the realistic assumption of nonzero measurement error, this negative relationship is observed in all areas of the parameter space explored.

### Simulated scenarios with true TL lengthening provide the best fit to empirical data

**Figure 4:**
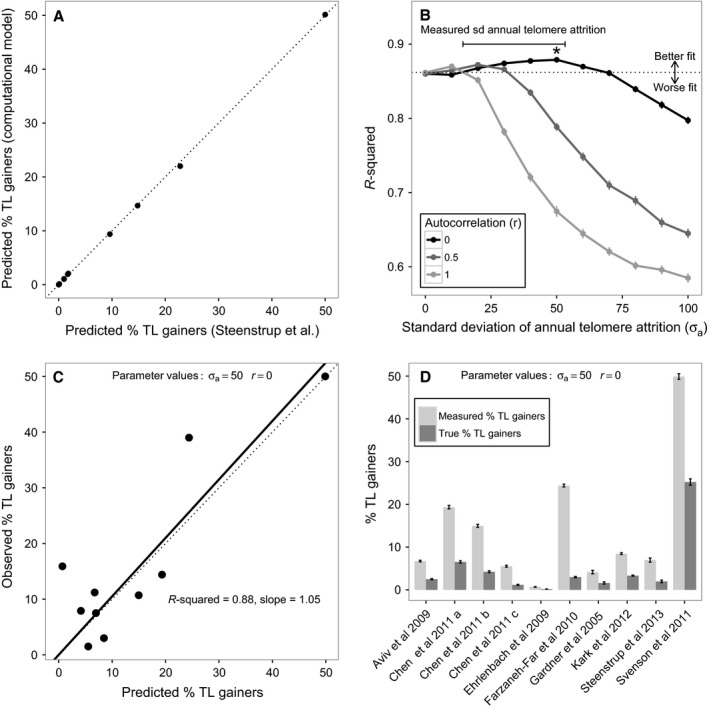
Simulated scenarios with true TL lengthening provide the best fit to empirical data on the observed percentage of TL gainers. Panels explore how well our computational model predicts the empirical data from the same 10 studies analysed by Steenstrup *et al*. (2013a; see Table S1, Supporting information). (A) Correlation between predicted percentage of TL gainers derived from Steenstrup *et al*.'s analytical approach and from our computational model with no true TL gainers (i.e. σ_*a*_ = 0 and *r *=* *0). The dotted line shows the expectation if the correlation is perfect. Results are based on assuming *n* = 10 000 individuals per study in the simulation. (B) The fit between the empirically observed percentage of TL gainers and the percentage of TL gainers predicted by the computational model for a range of different parameter value combinations. Results are based on simulations assuming the actual numbers of individuals in the empirical studies being modelled (*n *=* *50–635) and the reported number of interassay replicates of TL measurement (1 or 2; see Table S1, Supporting information for details). The points show the mean *r*‐squared values derived from 100 replicates of the simulation, and the error bars show the 95% confidence intervals for these means. The dotted line shows the fit of Steenstrup *et al*.'s model; points above this line correspond to parameter combinations in the computational model that predict the observed data better than the latter scenario, and points below this line correspond to parameter combinations that predict the observed data worse. The best‐fitting parameter combination (σ_*a*_ = 50 and *r *=* *0) is indicated with an asterisk (*). The range of values of the standard deviation of annual telomere attrition reported in papers from the Steenstrup *et al.'s* data set is shown with a bar; these comprise 14.3–14.9 (Kark *et al*., 2012), 29.4–53.2 (Chen *et al*., 2011), 27.4 (Steenstrup *et al*., 2013b) and 46.0 bp year^−1^ (Aviv *et al*., 2009). (C) Fig 1B replotted using the predictions derived from the best‐fitting parameter combination in the computational model in place of Steenstrup *et al*.'s predictions. (D) The predicted measured percentage of TL gainers and the predicted true percentage of TL gainers derived for each of the studies in the Steenstrup *et al*.'s data set using the best‐fitting parameter combination in the computational model. Bars show the mean ± 95% confidence intervals from 100 replicates of the simulation.

In order to verify that the computational model produces quantitatively similar predictions to those produced by Steenstrup *et al*.'s (2013a) simple analytical model, we set both σ_*a*_ and *r* to zero and simulated the percentage of TL gainers expected for each of the 10 studies for which they made predictions assuming 10 000 individuals per simulation. Reassuringly, there was a near‐perfect correlation between the predictions derived from the two different approaches (Pearson's correlation: *r* = 1.00, *n* = 10, *P* < 0.001; Fig. 4A).

Figure 4B shows the fit between the observed percentage of TL gainers and the model predictions for the 10 studies analysed by Steenstrup *et al*. (2013a) using a range of parameter value combinations to generate a set of telomere dynamics scenarios. Larger values of *r*‐squared indicate better model fit, and it is clear that in a large proportion of the parameter space, scenarios in which true telomere lengthening occurs (i.e. those in which σ_*a*_ > 0) provide a significantly better fit to the empirical data than the scenario with no lengthening (i.e. when σ_*a*_ = 0). Table 1 shows that of the 30 scenarios modelled with some true telomere lengthening, 10 (i.e. 33.33%) fitted the empirical data at least as well as Steenstrup *et al*.'s model. Therefore, Steenstrup *et al*.'s model is not unique in predicting the empirical data on telomere lengthening fairly well. Moreover, eight of the 30 scenarios with some true telomere lengthening (26.67%) provide a significantly better fit to the empirical data than Steenstrup *et al*.'s model.

**Table 1:** Summary of how the simulation results compare with the prediction from Steenstrup *et al*. (2013a) model for the range of scenarios of telomere dynamics explored in Fig. 4B

Autocorrelation (*r*)	Standard deviation of annual telomere attrition (σ_*a*_)										
	0	10	20	30	40	50	60	70	80	90	100
0	NS	–	*	*	*	*	*	NS	–	–	–
0.5	NS	NS	*	*	–	–	–	–	–	–	–
1.0	NS	*	–	–	–	–	–	–	–	–	–

‘NS’ indicates that the mean simulation result was not significantly different from Steenstrup *et al*. (2013a) model; ‘*’ indicates that the mean simulation result provided a significantly better fit than Steenstrup *et al*. (2013a) model; ‘–’ indicates that the mean simulation result provided a significantly worse fit than Steenstrup *et al*. (2013a) model. A significant difference in fit is defined as occurring when Steenstrup *et al*. (2013a) prediction falls outside the 95% confidence intervals of the mean prediction from the simulation.

The best‐fitting model of the empirical data occurs when the standard deviation of annual attrition is equal to approximately 50 bp and there is no autocorrelation in annual telomere attrition (i.e. σ_*a*_ = 50, *r *=* *0). Figure 4C shows how this best‐fitting model provides a closer fit to the empirical data from the 10 studies: unlike in Fig. 1B, the empirical data are no longer underpredicted by the theoretical model as evidenced by the smaller *r*‐squared value and slope closer to 1.00. Figure 4D shows the measured and true percentages of TL gainers in each study predicted by the best‐fitting model. The mean percentages of observed and true TL gainers are 14.12 ± 14.98% and 4.98 ± 7.33% (mean ± SD), respectively, for the 10 studies, and in all studies, the percentage of true TL gainers is greater than zero. Thus, although the majority of observed TL gain is due to measurement error, some true TL gain is predicted in all studies under this best‐fitting scenario.

## Discussion

To address the question of whether all the telomere lengthening observed in longitudinal epidemiological studies of TL is an artefact of measurement error, we created a computational model that allowed us to compare the predictions arising from a range of different assumptions regarding the true processes underlying observed telomere dynamics. Our analysis of the model yields clear answers to the two questions posed in the introduction. First, the prediction of a negative relationship between follow‐up period and observed percentage of TL gainers made by Steenstrup *et al*. (2013a) is not unique to the scenario they assume, in which all individuals show a constant rate of age‐related telomere attrition. Indeed, we found that this negative relationship is also predicted in many scenarios where there are both true lengthening error and measurement error, and indeed in some scenarios where there is no measurement error. Thus, the observation of a negative relationship between follow‐up period and observed percentage of TL gainers provides no information regarding the underlying telomere dynamics in operation. Second, the assumption that all individuals show a constant rate of age‐related telomere attrition does not yield the best quantitative fit to the empirical data on the percentage of TL gainers for the same 10 studies analysed by Steenstrup *et al*. (2013a). We found that a range of scenarios that include some true telomere lengthening yield a similar, and in some cases better, quantitative fit to the empirical data. Therefore, although we replicate Steenstrup *et al*. (2013a) basic results, we find no support for their conclusion that the apparent telomere lengthening observed in longitudinal studies is solely an artefact of measurement error as opposed to a real biological phenomenon. On the basis of our analysis, we conclude that the observed empirical data are insufficient to allow us to reject the hypothesis that true telomere lengthening occurs. Indeed, our data are more compatible with a model of telomere dynamics in which a small percentage (a mean of approximately 5%) of individuals exhibit true telomere lengthening. It is important to note that our analyses do not challenge Steenstrup *et al*. (2013a) conclusion that most telomere lengthening observed in longitudinal epidemiological studies is due to measurement error. Indeed, our analyses confirm this view, as shown by the discrepancies between the percentages of measured telomere length gainers and true telomere length gainers predicted by our best‐fitting model (Fig. 4D).

Our conclusion that telomere lengthening could be a real biological phenomenon observable in epidemiological studies is supported by recent *in vivo* longitudinal studies of leucocyte TL in humans in which observed telomere lengthening is unlikely to be attributable to measurement error. For example, in a population‐based observational study of 581 Costa Rican adults, Rehkopf *et al*. (2014) showed seasonal oscillation in leucocyte TL, with telomeres shortening in the wet season (when infections are most common) and lengthening in the dry season. The seasonal telomere lengthening observed in this study is unlikely to be attributable to measurement error because it is correlated across individuals in the sample. Even more compelling evidence for telomere lengthening comes from experimental studies in which subjects are exposed to some type of intervention. For example, Carulli *et al*. (2016) found significant leucocyte telomere lengthening after a 6‐month follow‐up in 37 obese adults that lost weight following insertion of bioenteric intragastric balloon. Similarly, García‐Calzón *et al*. (2014) found significant leucocyte telomere lengthening following a 2‐month intensive lifestyle intervention in 74 overweight/obese adolescents. Because both of these latter studies are experimental, the likelihood of the observed lengthening being an artefact of measurement error is extremely low. Indeed, measurement error should militate against finding significant intervention effects, because it will add noise to estimates of telomere attrition in both the experimental and control groups, reducing the power of the study to detect a treatment effect.

In addition to suggesting that some true telomere lengthening is compatible with the epidemiological data, our analyses additionally allow us to make inferences regarding the processes most likely to underlie human leucocyte telomere dynamics. Figure 4B suggests that the scenario of our model that best fits the empirical data is one in which the true standard deviation of annual attrition is approximately 40–50 bp and the autocorrelation in annual attrition is minimal. Sadly, to our knowledge, the data sets necessary to test these predictions are not currently available. To calculate the mean and standard deviation of annual attrition would require multiple annual longitudinal telomere measures from individual subjects using a precise measurement method, and this has not yet been done. Attempts to estimate the standard deviation of annual attrition from longer follow‐up periods have provided values ranging from 14 to 53 bp year^−1^ (Aviv *et al*., 2009; Chen *et al*., 2011; Kark *et al*., 2012; Steenstrup *et al*., 2013b). Encouragingly, this range corresponds to the values of σ_*a*_ that provide the best fit to the empirical data in our simulations (Fig. 4B). The suggestion arising from Fig. 4B that the autocorrelation in annual telomere attrition is minimal is on the face of it slightly puzzling. People's lifestyles are typically highly autocorrelated, and if, as is commonly assumed, lifestyle influences the rate of telomere attrition, then we might also expect annual telomere attrition to be highly autocorrelated. However, a test of this prediction awaits the collection of a suitable data set.

Our analyses emphasize the need to establish the variability in annual telomere attrition within individuals and how this is correlated between years in order to build accurate models of telomere dynamics. To this end, we recommend that more studies are required with repeated, short follow‐up periods, for example annual sampling of individuals for multiple years. To be of value in revealing short‐term telomere dynamics, such studies would need to minimize telomere measurement error. While the relative accuracy and precision of Southern blot (SB) and qPCR‐based methods are hotly debated (Martin‐Ruiz *et al*., 2015; Verhulst *et al*., 2015; Eisenberg, 2016), there is general agreement that measurement error can and should be reduced by further increasing the number of replicate measurements run per sample (Steenstrup *et al*., 2013a; Eisenberg, 2016). There has been recent discussion over the best statistic for characterizing measurement error and comparing it between SB and qPCR studies. CV is unlikely to be a valid measure, because the heteroscedasticity assumption of CV does not hold for qPCR and because SB measures are not on a ratio scale (Eisenberg, 2016; Verhulst *et al*., 2016). In the current paper, we used CV, because this is the only statistic reported in the existing empirical literature on telomere dynamics and because it was important for us to exactly replicate the methods used by Steenstrup *et al*. (2013a). Future empirical studies should consider reporting alternative statistics such as the intraclass correlation coefficient (Verhulst *et al*., 2016). Finally, we urge the publication of raw telomere length data, because this would facilitate the use of published data sets in future meta‐analytic studies in which the summary statistics required differ from those provided by the authors.

The current paper highlights the need for explicit process‐level models in human telomere dynamics. The value of such models is twofold. First, creating a process‐level model has the virtue of forcing us to be explicit about the assumptions that we are making regarding the underlying processes. Second, process‐level models can be used to generate quantitative predictions of the patterns expected in the empirical data under different scenarios. Such predictions can then be tested against the empirical data. It is important to consider multiple plausible process‐level models and to use the data to choose between these (for an excellent discussion of this point, see McElreath, 2016 p. 5–7). As we have seen in the current paper, it is often the case that multiple different process‐level models will produce similar predictions of patterns in the data, and the task is therefore to find predictions that allow us to discriminate between different models. Steenstrup *et al*. (2013a) made an important advance in assuming an explicit process‐level model of telomere dynamics, namely that all individuals experience age‐related telomere attrition, and exploring the predictions of this model under the assumption of measurement error. Here, we have extended their approach by comparing multiple models of telomere dynamics and showing that the model producing the best fit to the current empirical data on the observed percentage of TL gainers is actually one that includes some true telomere lengthening.

## Description of the computational model

The model generates annual values of true TL for a sample of individuals followed over multiple years given a set of assumptions about telomere dynamics. Table 2 summarizes the key parameters of the model and gives the default values assumed in our simulations.

**Table 2:** Summary of the key parameters in the model

Parameters	Description	Default value assumed or range of values explored
*n*	Number of individuals in the simulation	10 000
μ_*b*_	Mean baseline TL	7000 bp
σ_*b*_	Standard deviation of baseline TL	700 bp
μ_*a*_	Mean telomere attrition per year	30 bp year^−1^
σ_*a*_	Standard deviation of annual telomere attrition	0, 50 and 100 bp
*r*	Autocorrelation between telomere attrition in successive years	0, 0.5 and 1
*CV*	Coefficient of variation of measurement error	2, 4 and 8%
*y*_max_	Maximum follow‐up period	15 years

The simulation of true TLs proceeds as follows. In the first step, a sample of baseline TLs is generated for *n* individuals. Baseline TLs are independently generated random samples from a normal distribution with mean μ_*b*_ and standard deviation σ_*b*_. In the second step, annual telomere attrition is calculated for each individual. In the first follow‐up year, this annual telomere attrition is drawn from a normal distribution with mean μ_*a*_ and standard deviation σ_*a*_. Setting σ_*a*_ equal to a number greater than zero creates the possibility for annual attrition to be either greater than or less than μ_*a*_, and hence allows us to model the possibility that some individuals’ TLs can increase from baseline. In follow‐up years after the first, annual attrition is calculated according to Eqn (1):

(1) $${attrition}_{y}\;=\;r\;\cdot \;{attrition}_{y-1}\;+\;\sqrt{\left(1-r^{2}\right)}\;N\left(\frac{\left(1-r\right)}{\sqrt{\left(1-r^{2}\right)}}\mu_{a},\mu_{a}\right)$$

where *attrition*_*y*_ is the telomere attrition in the current year, *attrition*_*y‐1*_ is the telomere attrition in the previous year, *r* is a parameter controlling the autocorrelation between attrition in subsequent years, and *N(i, j)* designates an independently generated random sample from a normal distribution with a mean *i* and standard deviation *j*. Eqn (1) generates annual attrition with a mean in every year across individuals equal to μ_*a*_, a standard deviation equal to σ_*a*_ and a correlation of *r* with attrition in the previous year (see Appendix S1, Supporting information for a proof of these statements). Setting *r* equal to a number greater than zero generates autocorrelation in annual attrition and hence allows us to model the possibility that individuals experience consistently different rates of annual telomere attrition. If *r* is set equal to zero, the first term of Eqn (1) disappears, and annual attrition is calculated independently of its value in the previous year, as in the first follow‐up year; if *r* is set to a value of one, annual attrition is identical to that experienced in the first follow‐up year in all subsequent years. In the third step, the annual attrition is subtracted from the current TL for each individual to generate a new value for current TL and this latter value is saved. Steps two and three are repeated until the maximum follow‐up period *y*_*max*_ is reached.

The above model yields an array (with dimensions *n* by *y*_*max*_ + 1) that gives the true TL for each individual in each year of the simulation from baseline (year 0) to *y*_*max*_. For each follow‐up year, these data are used to calculate the percentage of individuals in the simulation whose true TL increases relative to the baseline value, referred to as the percentage of true TL gainers.

Measurement error is introduced by assuming that measured TL is an independently generated random sample from a normal distribution with the mean equal to the true TL and the standard deviation equal to the true TL * *CV*/100. This yields a second array (with dimensions *n* by *y*_*max*_ + 1) that gives the measured TL for each individual in each year of the simulation. For each follow‐up year, these data are used to calculate the percentage of individuals in the simulation whose measured TL increases relative to the baseline measurement, referred to as the percentage of observed TL gainers. Note that in our simulations, each true TL is ‘measured’ only once, but the improved precision arising from replicate measures can be estimated by simply reducing the value of *CV* assumed (see Steenstrup *et al*., 2013a).

To generate predictions from our model for the same 10 empirical studies considered by Steenstrup *et al*. (2013a: Aviv *et al*., 2009; Chen *et al*., 2011; Ehrlenbach *et al*., 2009; Farzaneh‐Far *et al*., 2010; Gardner *et al*., 2005; Kark *et al*., 2012; Steenstrup *et al*., 2013b; Svenson *et al*., 2011), we re‐ran our model using the values for *n*, follow‐up period (*y*_*max*_), μ_*b*_, σ_*b*_, μ_*a*_, interassay replicate number (rep) and *CV* for each study given in Table 1 of their paper (reproduced as Table S1, Supporting information of the current paper). As Steenstrup *et al*. (2013a) did not report σ_*b*_ for the studies they analysed, we returned to the original papers to extract these values. For the studies that did not report σ_*b*_ (Ehrlenbach *et al*., 2009; Svenson *et al*., 2011; Steenstrup *et al*., 2013b), we assumed a standard deviation of 700 bp (because this was approximately the mean of the values obtained for the other studies). Following Steenstrup *et al*. (2013a) methods, where the number of interassay replicates reported for a study was 2 (as opposed to only 1), we reduced the value of *CV* assumed in our simulations (*CV* = *CV*/$\sqrt{2}$ to reflect the increased precision of measurement obtained with two replicates. To quantify how well alternative scenarios of our model predicted the percentage of observed TL gainers in the 10 studies, for each scenario we calculated the r‐squared value for a linear regression (forced through the origin) for the observed values on the predicted values. The scenario assumed by Steenstrup *et al*. (2013a) was modelled by setting both σ_*a*_ and *r* equal to zero, whereas scenarios with true telomere lengthening were modelled by setting σ_*a*_ to values greater than zero. Finally, to locate the parameter values that yielded the best model fit (i.e. the largest r‐squared value), we conducted an exploration of the parameter space for both σ_*a*_ and *r*. We ran 100 replicates of this simulation in order to generate 95% confidence intervals for the mean results.

The computational model was implemented in the R language for statistical computing (R Core Team 2013). R scripts for running the model and generating all the results contained in this paper are available as Supporting Information (R script S1 and R script S2).

## Funding

This work was funded by the National Centre for the Replacement Refinement and Reduction of Animals in Research (Project Grant NC/K000802/1 to MB) and the European Research Council (AdG 666669 to DN). We thank Abraham Aviv, Troels Steenstrup, Peter Adams and two anonymous referees for helpful comments on the manuscript.

## Author contributions

MB conceived the paper, built and analysed the computational model and wrote the first draft of the manuscript. DN advised on all aspects of the paper, derived Eqn (1) and wrote Appendix S1 (Supporting information).

## Conflict of interest

None declared.

## Supporting information


**Appendix S1.** Mathematical proof of Eqn (1) for determining the annual telomere attrition.

**Fig. S1** Descriptive statistics for the simulated distribution of true TLs in each year. (A) Mean and (B) standard deviation of true TL at each year of follow‐up for the same nine scenarios depicted in Fig. 2. Results are based on 10 000 individuals per simulation.

**Table S1.** Data from the empirical studies analysed by Steenstrup *et al*. (2013a) and in the current paper (required by R script S2).

**R script S1.** An R function to generate and plot true and measured TLs.

**R script S2.** An R script to generate the results reported in the current paper using R script S1.
