# Supplementary material for: The telomere lengthening conundrum – it could be biology
Source: Aging Cell. 2016 Dec 12;16(2):312–9. doi: 10.1111/acel.12555 (PMC5334537; doi:10.1111/acel.12555)
Supplement: Supplementary file 1 — Appendix S1. Mathematical proof of Eqn (1) for determining the annual telomere attrition. [file ACEL-16-312-s001.pdf]

Appendix S1 for 'The telomere lengthening conundrum – it could be biology'

As part of our model, it is necessary to simulate annual telomere attrition values for each year that are correlated with the previous year's values with correlation coefficient *r*, whilst maintaining the required mean annual telomere attrition μ_a and required standard deviation of annual attrition σ_a. Here, we prove that appropriate values can be generated using the formula:

$$attrition_y = r \cdot attrition_{y-1} + \sqrt{(1-r^2)}\, X \qquad (1)$$

In this expression, $attrition_y$ is the attrition values for the current year; $attrition_{y-1}$ is the attrition values for the previous year, and X is an independently generated random variable with distribution $X \sim N(\frac{(1-r)}{\sqrt{(1-r^2)}}\mu_a, \sigma_a)$. To prove that (1) is the appropriate formula, we must show that it has expected value μ_a, standard deviation σ_a, and that the correlation between $attrition_{y-1}$ and $attrition_y$ is *r.*

First, the expected value of $attrition_y$. By linearity of expectation:

$$\begin{aligned} E(attrition_y) &= E(r \cdot attrition_{y-1} + \sqrt{(1-r^2)}\, X) \\ &= r \cdot E(attrition_{y-1}) + \sqrt{(1-r^2)}\, E(X) \\ &= r\mu_a + \sqrt{(1-r^2)}\, \frac{(1-r)}{\sqrt{(1-r^2)}}\mu_a \\ &= r\mu_a + (1-r)\mu_a \\ &= \mu_a \end{aligned}$$

as required.

Next, we derive the variance of $attrition_y$. Because of the independence of $attrition_{y-1}$ and X,

$$\begin{aligned} \mathrm{V}(attrition_y) &= \mathrm{V}(r \cdot attrition_{y-1} + \sqrt{(1-r^2)}\, X) \\ &= r^2 \mathrm{V}(attrition_{y-1}) + (1-r^2)V(X) \\ &= r^2\sigma_a^2 + (1-r^2)\sigma_a^2 \\ &= \sigma_a^2 \end{aligned}$$

Hence, the standard deviation of $attrition_y$ is σ_a.

Finally, the correlation between $attrition_y$ and $attrition_{y-1}$ is derived as follows. From the definition of correlation:

$$\begin{aligned}\mathrm{Cor}(attrition_y, attrition_{y-1}) &= \frac{\mathrm{Cov}(attrition_y, attrition_{y-1})}{\sqrt{\mathrm{V}(attrition_y)\mathrm{V}(attrition_{y-1})}} \\ &= \frac{\mathrm{Cov}(attrition_y, attrition_{y-1})}{\sigma_a^2}\end{aligned}$$

Since X is independent of $attrition_{y-1}$, $\mathrm{Cov}\left(attrition_{y-1}, X\right) = 0$. Moreover, $\mathrm{Cov}\left(attrition_{y-1}, r \cdot attrition_{y-1}\right) = r \cdot V(attrition_{y-1}) = r\sigma_a^2$. Hence:

$$\begin{aligned}\mathrm{Cov}\left(attrition_y, attrition_{y-1}\right) &= \mathrm{Cov}(attrition_{y-1}, r \cdot attrition_{y-1} + \sqrt{(1-r^2)}\, X) \\ &= r \cdot V(attrition_{y-1}) \\ &= r\sigma_a^2\end{aligned}$$

Thus, as required:

$$\begin{aligned}\mathrm{Cor}\left(attrition_y, attrition_{y-1}\right) &= \frac{r\sigma_a^2}{\sigma_a^2} \\ &= r\end{aligned}$$
