# Supplementary figures and images for: The telomere lengthening conundrum – it could be biology
Source: Aging Cell. 2016 Dec 12;16(2):312–9. doi: 10.1111/acel.12555 (PMC5334537; doi:10.1111/acel.12555)

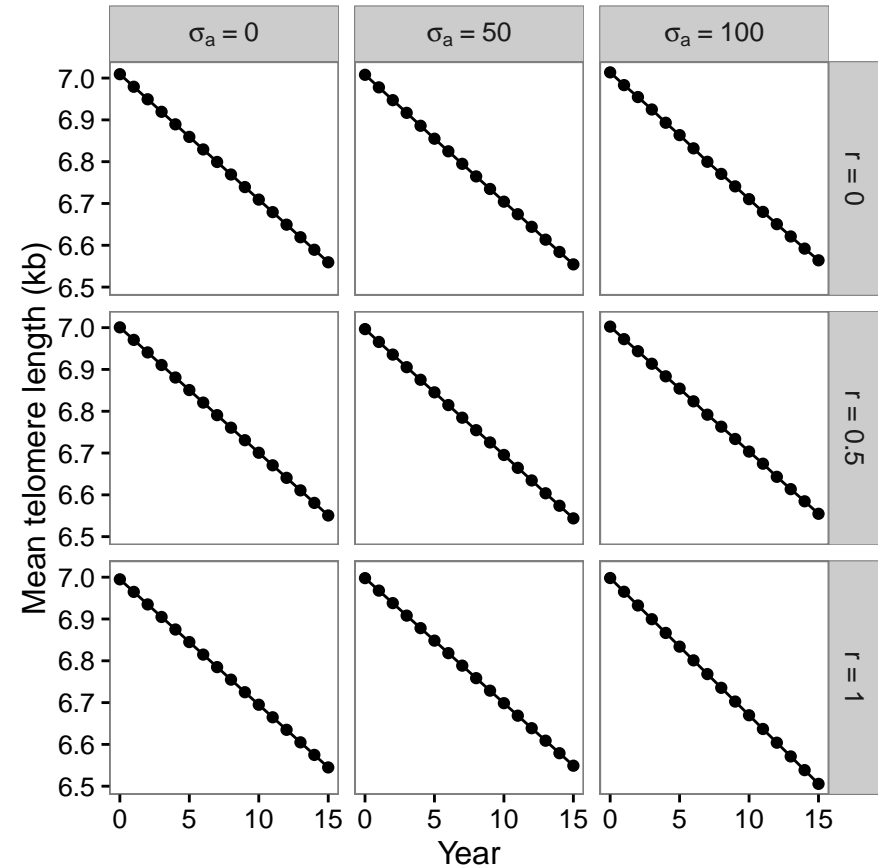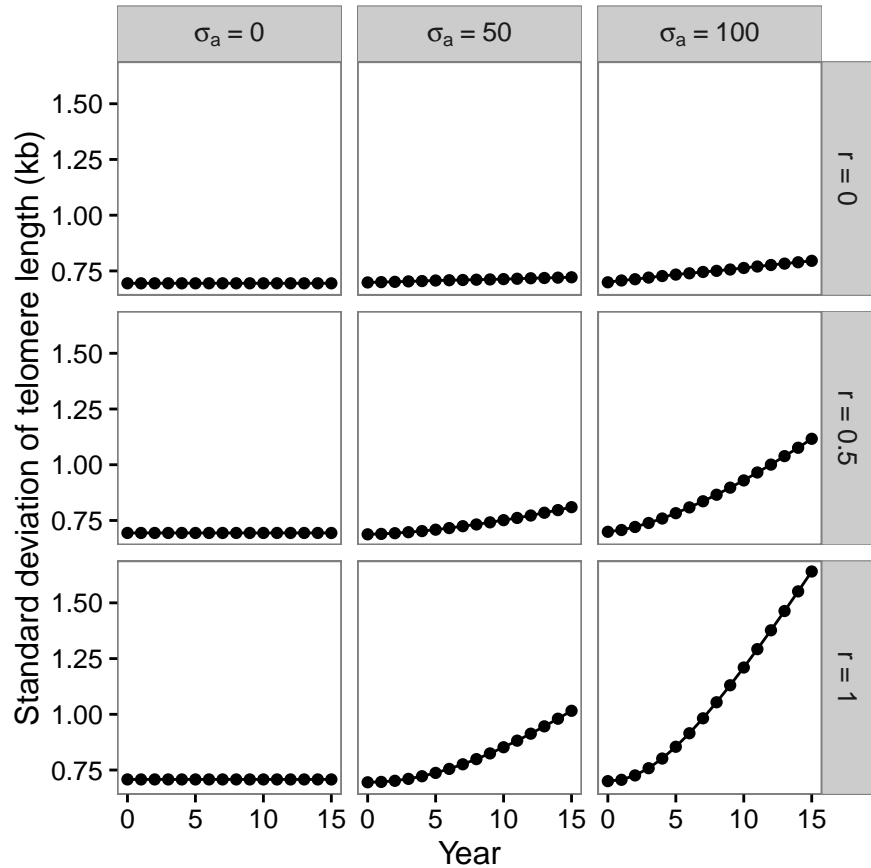

Supplement: Supplementary file 2 — Fig. S1 Descriptive statistics for the simulated distribution of true TLs in each year. (A) Mean and (B) standard deviation of true TL at each year of follow‐up for the same nine scenarios depicted in Fig. 2. Results are based on 10 000 individuals per simulation. [file ACEL-16-312-s002.pdf]
